# Effect of 2-Weeks Ischemic Preconditioning on Exercise Performance: A Pilot Study

**DOI:** 10.3389/fspor.2021.646369

**Published:** 2021-06-14

**Authors:** Daichi Tanaka, Tadashi Suga, Kento Shimoho, Tadao Isaka

**Affiliations:** Faculty of Sport and Health Science, Ritsumeikan University, Kusatsu, Japan

**Keywords:** peak O_2_ consumption, local endurance, O_2_ dynamics, muscle strength, muscle hypertrophy

## Abstract

An acute bout of ischemic preconditioning (IPC) has been reported to increase exercise performance. Nevertheless, the ineffectiveness of acute IPC on exercise performance has also been reported. Similarly, the effect of a shot-term intervention of IPC on exercise performance remains controversial in previous studies. In this study, we examined the effects of short-term IPC intervention on whole and local exercise performances and its-related parameters. Ten healthy young males undertook a 2-weeks IPC intervention (6 days/weeks). The IPC applied to both legs with three episodes of a 5-min ischemia and 5-min reperfusion cycle. Whole-body exercise performance was assessed by peak O_2_ consumption (VO_2_: VO_2__peak_) during a ramp-incremental cycling test. Local exercise performance was assessed by time to task failure during a knee extensor sustained endurance test. A repeated moderate-intensity cycling test was performed to evaluate dynamics of pulmonary VO_2_ and muscle deoxygenation. The knee extensor maximal voluntary contraction and quadriceps femoris cross-sectional area measurements were performed to explore the potentiality for strength gain and muscle hypertrophy. The whole-body exercise performance (i.e., VO_2__peak_) did not change before and after the intervention (*P* = 0.147, Power = 0.09, Effect size = 0.21, 95% confidence interval: −0.67, 1.09). Moreover, the local exercise performance (i.e., time to task failure) did not change before and after the intervention (*P* = 0.923, Power = 0.05, Effect size = 0.02, 95% confidence interval: −0.86, 0.89). Furthermore, no such changes were observed for all parameters measured using a repeated moderate-intensity cycling test and knee extensor strength and quadriceps femoris size measurements. These findings suggest that a 2-weeks IPC intervention cannot increase whole-body and local exercise performances, corresponding with ineffectiveness on its-related parameters in healthy young adults. However, the statistical analyses of changes in the measured parameters in this study showed insufficient statistical power and sensitivity, due to the small sample size. Additionally, this study did not include control group(s) with placebo and/or nocebo. Therefore, further studies with a larger sample size and control group are required to clarify the present findings.

## Introduction

Previous studies have reported that an acute bout of ischemic preconditioning (IPC) increases whole-body and local exercise performances (de Groot et al., 2010; Crisafulli et al., 2011; Kido et al., 2015; Tanaka et al., 2016; Paradis-Deschênes et al., 2018), mainly assessed with peak O_2_ consumption (VO_2_; VO_2__peak_) and local endurance time. Nevertheless, a meta-analysis by Salvador et al. (2016) showed that this positive effect had a small effect (i.e., effect size = 0.43). Furthermore, other studies have reported the ineffectiveness of acute IPC on exercise performance (Clevidence et al., 2012; Hittinger et al., 2015; Tocco et al., 2015). Therefore, the effect of acute IPC on exercise performance remains controversial in previous studiess.

Several studies have reported that short-term intervention of IPC increases whole-body exercise performance (Lindsay et al., 2017; Jeffries et al., 2018, 2019). In contrast, other studies have reported the ineffectiveness of short-term IPC intervention on whole-body exercise performance (Lindsay et al., 2018; Slysz and Burr, 2019). Therefore, in addition to acute IPC, the effect of short-term IPC intervention on whole-body exercise performance also remains controversial.

Lindsay et al. (2017) reported that a 1-week IPC intervention increased whole-body exercise performances, including VO_2__peak_ during a ramp-incremental cycling test. Jeffries et al. (2019) also reported positive effect of a 1-week IPC intervention on endurance time, but not VO_2__peak_, during a ramp-incremental cycling test. Additionally, another study by Jeffries et al. (2018) reported that a 1-week IPC intervention increased oxidative capacity in the skeletal muscle. The skeletal muscle oxidative capacity is related to local exercise performance (Okita et al., 1998; Homma et al., 2009), such as endurance time during localized exercise (Homma et al., 2009). However, the effect of short-term IPC intervention on local exercise performance remains unknown.

Paradis-Deschênes et al. (2016) reported that acute IPC increased peak force output during maximal voluntary contraction (MVC) throughout repeated knee extensor exercises. Their finding suggests that short-term IPC intervention may have potential to increase muscle strength. Additionally, because of the close relationship between muscle strength and size (Hori et al., 2020; Tottori et al., 2020), short-term IPC intervention may also potentially increase muscle size. An increase in whole-body exercise performance (e.g., VO_2__peak_) induced by long-term exercise training may be associated with increased muscle strength and size (Frontera et al., 1990; Salvadego et al., 2013). Therefore, positive effect of short-term IPC intervention on whole-body exercise performance may be at least partially due to increased muscle strength and/or size. However, the effects of short-term IPC intervention on these muscle adaptations remain poorly understood.

Lindsay et al. (2017) found that whole-body exercise performance (e.g., $\dot{\text{V}}$O_2peak_) further increased by a week after completion of a 1-week IPC intervention compared to 48 h after the completion of this intervention. Their finding suggests that increasing the IPC intervention period (e.g., 2 weeks) may be effective in enhancing its positive effects. Therefore, in this study, we aimed to determine the effects of a 2-weeks IPC intervention on whole-body and local exercise performances and its-related parameters.

## Methods

### Subjects

Ten healthy, young males (age: 23 ± 1 years) participated in this study. The subjects were recreationally active, but did not participate in specific physical training program within 3 years. None of the subjects had contraindications to magnetic resonance imaging (MRI). All subjects were informed about the experimental procedures and potential risks and gave written consent to participate in the study. This study was approved by the Ethics Committee of Ritsumeikan University (IRB-2017-034).

### Experimental Design

**Figure 1:**
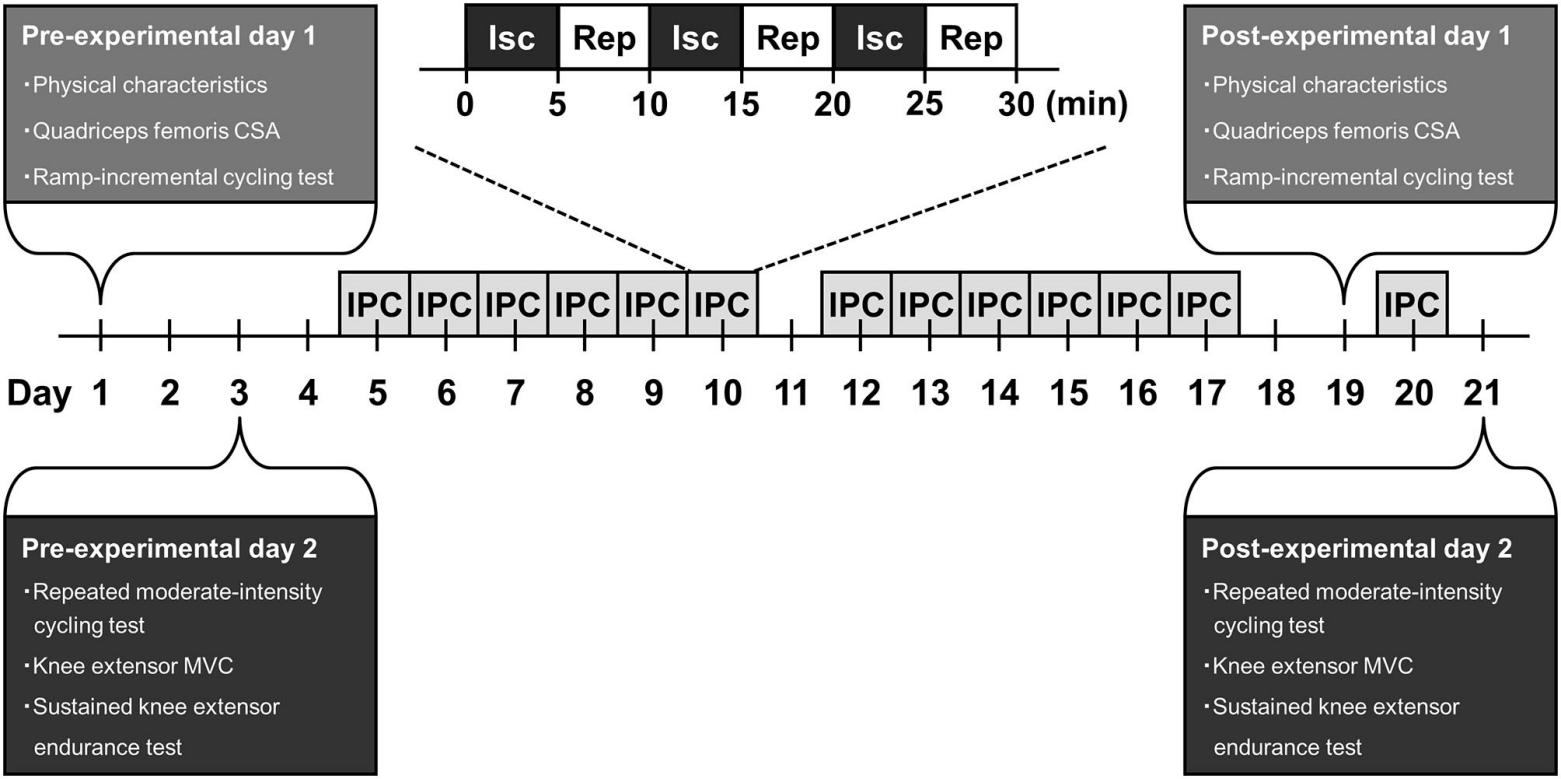
A schematic representation of experimental design. CSA, cross-sectional area; MVC, maximal voluntary contraction; Isc, ischemia; Rep, reperfusion; IPC, ischemic preconditioning.

A schematic representation of the experimental design of this study is shown in Figure 1. Subjects were required to visit the laboratory on 17 occasions over a 3-weeks period. Prior to the 2-weeks IPC intervention, the subjects visited the laboratory to perform required measurements throughout 2 days. The required measurements for each experimental day completed in the morning. The subjects were instructed to arrive at the laboratory in overnight fasted state and to avoid strenuous physical activity in 24 hours before the experiments. The subjects were also instructed to abstain from caffeine and alcohol intake for 6 and 24 h before the experiments, respectively.

On the first experimental day, physical characteristics and quadriceps femoris cross-sectional area (CSA) of the subjects was measured. Then, the subjects performed a ramp-incremental cycling test. Forty-eight hours after the day, in the second experimental day, the subjects performed a repeated moderate-intensity cycling test. Thirty minutes after this test was completed, the knee extensor MVC of the subjects was measured. Thereafter, the subjects performed a sustained knee extensor endurance test.

Two days after the second experimental day, the subjects undertook a 2-weeks IPC intervention with 6 days/week, which is a total of 12 sessions. An IPC session of each day comprised of three episodes of a 5-min ischemia and 5-min reperfusion cycle to both legs. Cuffs for IPC were placed proximally around the upper thighs of the subjects in a supine position and inflated to 220 mmHg. The 12 sessions of IPC throughout 2-weeks intervention period in all subjects were performed at approximately the same time (± 1 h) in the morning every day.

Forty-eight hours after the final IPC session of the 2-weeks intervention, the subjects repeated same measurements on the first experimental day before this intervention. On the next day, the subjects returned to the laboratory to undergo one IPC session, as aimed to sustain the effect on this IPC intervention. Again, 24 h after the last IPC session, the subjects repeated same measurements on the second experimental day before the IPC intervention.

### Quadriceps Femoris CSA Measurement

The detailed method of the quadriceps femoris CSA measurement using MRI has been described in our previous studies (Miyake et al., 2017; Hori et al., 2020; Tottori et al., 2020, 2021). In brief, the MRI measurement of the quadriceps femoris CSA was performed using a 1.5-T magnetic resonance system (Signa HDxt; GE Medical Systems, WI, USA). The CSA of the right quadriceps femoris, including the rectus femoris, vastus intermedius, vastus lateralis, and vastus medialis, was obtained at the proximal 50% of the thigh length (Miyake et al., 2017; Tottori et al., 2021). In our previous study, we have reported the reproducibility of the mid-thigh quadriceps femoris CSA on two separate days in 14 healthy young males (Miyake et al., 2017). The interclass correlation coefficient (ICC) of the mid-thigh quadriceps femoris CSA on the 2 days was 0.968, which can be considered as excellent value (Koo and Li, 2016).

### Ramp-Incremental Cycling Test

The detailed method of the ramp-incremental cycling test has been described in our previous studies (Kido et al., 2015, 2018). In brief, subjects performed 5 min of a warm-up cycling at 30 watts/min on a cycling ergometer (Ergomedic 828E; Monark Exercise AB, Vansbro, Swede), after which the workload increased by 30 watts/min until task failure. The subjects were asked to maintain a cadence of 60 rpm. During this test, breath-by-breath pulmonary gas exchange data were collected by using a gas analyzer (AE-310S; Minato Medical Science, Osaka, Japan) and averaged every 10 s. The gas exchange threshold was determined using the V slope method (Beaver et al., 1986). The VO_2__peak_ was determined as the highest 30-s mean value attained prior to exhaustion.

### Repeated Moderate-Intensity Cycling Test

The repeated moderate-intensity cycling test was consisted of the following protocol; 4-min low-intensity cycling at 30 watts/min (i.e., the first low-intensity period), 4-min moderate-intensity cycling at 90% of the gas exchange threshold (i.e., the first moderate-intensity period), 4-min active rest with a low-intensity cycling at 30 watts/min, 4-min low-intensity cycling at 30 watts/min (i.e., the second low-intensity period), and 4-min moderate-intensity cycling at 90% of the gas exchange threshold (i.e., the second moderate-intensity period). Subjects were asked to maintain a cadence of 60 rpm throughout this test. During this test, breath-by-breath pulmonary VO_2_ was recorded continuously using a gas analyzer. The tissue deoxy-hemoglobin/myoglobin, which is a reliable estimator of intramuscular oxygenation state, of the quadriceps vastus lateralis was also recorded continuously using a near-infrared spectroscopy (NIRO 200; Hamamatsu Photonics, Shizuoka, Japan). The detailed methods for calculating the pulmonary VO_2_ and muscle deoxygenation dynamics have been described in our previous studies (Kido et al., 2015, 2018). In our previous study, we have reported the reproducibility of pulmonary VO_2_ and muscle deoxygenation dynamics on two separate days in 10 healthy young males (Kido et al., 2015). The ICCs of variables (i.e., time delay, time constant, and mean response time) of pulmonary VO_2_ and muscle deoxygenation dynamics during a single session of moderate-intensity cycling on the 2 days were excellent values (e.g., 0.983 and 0.954, respectively, for each mean response time).

### Knee Extensor MVC Measurement

The detailed method of the knee extensor MVC measurement has been described in our previous studies (Tanaka et al., 2016, 2020; Hori et al., 2020; Tottori et al., 2020). In brief, the knee extensor MVC was measured using a BIODEX dynamometer system (BIODEX system 3; BIODEX Medical, Shirley, NY, USA). The two knee extensor MVC trials were performed each for 3 s with a 1-min rest period. If the difference in the peak torque values between the two trials was more than 5% of the highest value, additional trials were performed until this was corrected. The highest peak torque of the two trials or more than two trials was assessed as the knee extensor MVC. In the reproducibility of the knee extensor MVC, we calculated the ICC on two separate days in 14 healthy young males, as obtained in our previous study (Tanaka et al., 2020). The ICC of the knee extensor MVC on the 2 days showed an excellent value of 0.974.

### Sustained Knee Extensor Endurance Test

The detailed methods for performing the sustained knee extensor endurance test and for analyzing the quadriceps femoris electromyographic activity have been described in our previous studies (Tanaka et al., 2016, 2020). In brief, a target torque of this test in subjects was determined as 20% of their knee extensor MVC. The subjects were required to match the target torque as displayed on the monitor and verbally encouraged to sustain the torque for as long as possible. The sustained knee extensor isometric contraction was continued to task failure, which was defined as a declination in torque to <90% of the 20% MVC target torque for more than 5 s, despite maximum effort. During this test, torque signals were sampled at 100 Hz to calculate exercise endurance (i.e., time to task failure) and mean torque output. Electromyographic activities in three quadriceps femoris muscles, including the vastus lateralis, vastus medialis, and rectus femoris, were also recorded continuously using an electromyograph (MQ-Air; Kissei Comtech, Nagano, Japan).

### Statistical Analysis

Data are expressed as mean ± standard deviation. Comparisons of measured variables before and after a 2-weeks IPC intervention were performed using a paired Student's *t*-test. The level of significance was set at *P* < 0.05. The power (β) was calculated to determine the level of statistical power of change in measured variable before and after the intervention. The Cohen's *d* effect size (ES) was calculated to determine the magnitude of change in measured variable before and after the intervention (Cohen, 1992). The 95% confidence interval (CI) of the ES was calculated to determine its statistical sensitivity. All statistical analyses were conducted using SPSS (version 19.0; International Business Machines Corp, NY, USA) or G^*^Power software.

## Results

Physical characteristics did not change before and after 2-weeks IPC intervention (body height: 171.9 ± 4.0 vs. 172.0 ± 4.2 cm, *P* = 0.418, Power = 0.05, ES = 0.03, 95% CI: −0.85, 0.91: body weight; 65.9 ± 10.0 vs. 66.0 ± 10.0 kg, *P* = 0.936, Power = 0.05, ES = 0.00, 95% CI: −0.87, 0.88).

**Figure 2:**
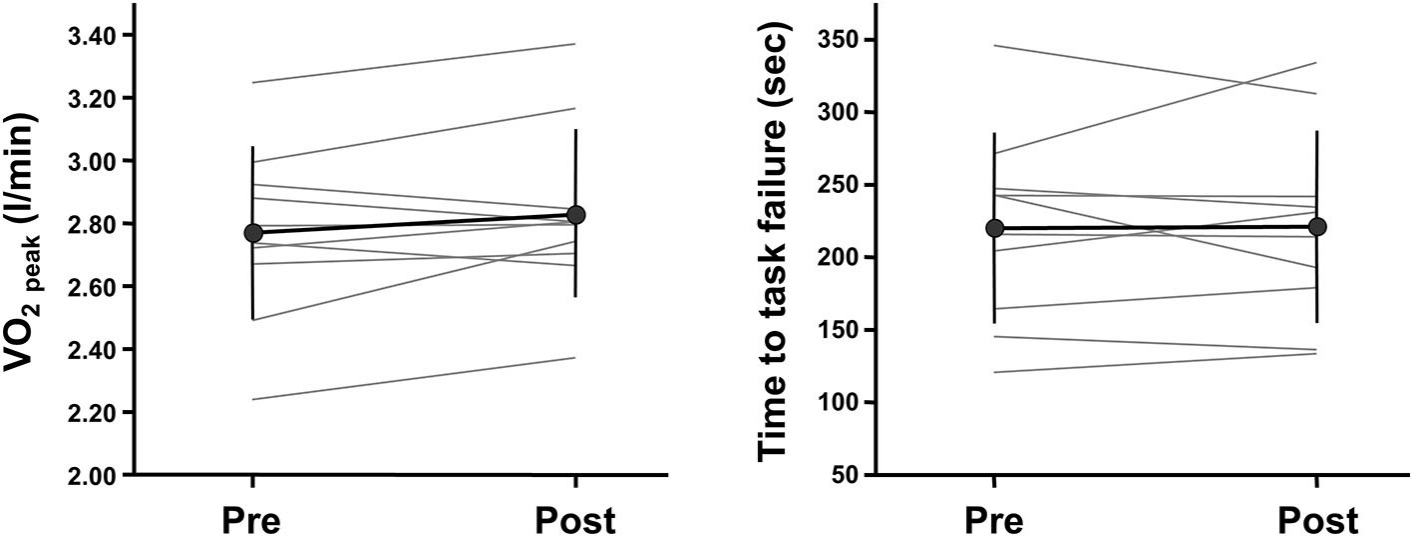
Individual changes in whole-body and local exercise performances before and after a 2-weeks ischemic preconditioning intervention. Whole-body and local exercise performances are assessed with peak oxygen consumption (VO_2 peak_) during a ramp-incremental cycling test and time to task failure during a knee extensor sustained endurance test, respectively.

Changes in measured variables during ramp incremental cycling and repeated moderate-intensity cycling tests before and after 2-weeks IPC intervention are summarized in Table 1. VO_2 peak_ during a ramp incremental cycling test did not change before and after the intervention (Figure 2; *P* = 0.147, Power = 0.09, ES = 0.21, 95% CI: −0.67, 1.09). Additionally, all variables of pulmonary VO_2_ and muscle deoxygenation dynamics during a repeated moderate-intensity cycling test did not change before and after the intervention (*P* = 0.213–0.878, Power = 0.05 to 0.23, ES = −0.04 to 0.43, for all). Furthermore, other variables measured during ramp incremental cycling and repeated moderate-intensity cycling tests did not change significantly before and after the intervention (see Table 1).

**Table 1:** Changes in measured variables during ramp-incremental cycling and repeated moderate-intensity cycling tests before and after a 2-weeks ischemic preconditioning intervention.

	**Pre**	**Post**	***P*-value**	**Power (β)**	**Effect size (*d*)**	**95% confidence interval**
**Ramp incremental cycling test**						
Gas exchange threshold, l/min	1.61 ± 0.20	1.62 ± 0.17	0.615	0.05	0.06	−0.82, 0.94
Peak VO_2_, l/min	2.77 ± 0.28	2.83 ± 0.27	0.147	0.09	0.21	−0.67, 1.09
Peak exercise load, watt/min	273.0 ± 17.0	279.0 ± 24.7	0.168	0.12	0.28	−0.60, 1.16
Peak heart rate, bpm	187.3 ± 7.5	189.4 ± 8.2	0.265	0.12	0.27	−0.61, 1.15
**Repeated moderate-intensity cycling test**						
End-exercise VO_2_, l/min	1.66 ± 0.18	1.66 ± 0.17	0.922	0.05	−0.01	−0.89, 0.86
Exercise load, watt/min	124.5 ± 12.3	126.0 ± 10.5	0.343	0.07	0.13	−0.75, 1.01
End-exercise heart rate, bpm	136.5 ± 10.3	140.0 ± 10.8	0.273	0.15	0.33	−0.56, 1.21
**Pulmonary VO**_**2**_ **dynamics**						
Amplitude, l/min	1.00 ± 0.16	1.02 ± 0.15	0.343	0.08	0.17	−0.71, 1.04
Time delay, sec	16.4 ± 4.6	18.3 ± 6.2	0.213	0.17	0.35	−0.53, 1.23
Time constant, sec	42.8 ± 9.0	42.3 ± 14.0	0.878	0.05	−0.04	−0.92, 0.83
Mean response time, sec	59.2 ± 8.8	60.6 ± 18.0	0.747	0.06	0.10	−0.78, 0.98
**Muscle deoxygenation dynamics**						
Amplitude,%	20.6 ± 9.8	25.8 ± 13.7	0.313	0.23	0.43	−0.45, 1.32
Time delay, sec	11.6 ± 2.8	12.4 ± 5.4	0.640	0.08	0.19	−0.69, 1.06
Time constant, sec	14.5 ± 5.5	17.0 ± 13.0	0.519	0.11	0.25	−0.63, 1.13
Mean response time, sec	26.1 ± 7.4	29.4 ± 14.9	0.455	0.12	0.28	−0.60, 1.16

*Values are presented as mean ± standard deviation. VO_2_; O_2_ consumption*.

Changes in local muscle variables before and after 2-weeks IPC intervention are summarized in Table 2. The quadriceps femoris CSA and knee extensor MVC did not change before and after the intervention (*P* = 0.730 and 0.217, Power = 0.05 and 0.07, ES = −0.02 and 0.13, respectively). Additionally, endurance (i.e., time to task failure) during a sustained knee extensor endurance test did not change before and after the intervention (Figure 2; *P* = 0.923, Power = 0.05, ES = 0.02, 95% CI: −0.86, 0.89). Other variables measured during this test also did not change significantly before and after the intervention (see Table 2).

**Table 2:** Changes in measured local muscle variables before and after a 2-weeks ischemic preconditioning intervention.

	**Pre**	**Post**	***P*-value**	**Power (β)**	**Effect size (*d*)**	**95% confidence interval**
**Quadriceps femoris CSA, cm**^**2**^	71.9 ± 10.3	71.6 ± 11.9	0.730	0.05	−0.02	−0.90, 0.86
**Knee extensor MVC, Nm**	241.7 ± 48.0	247.6 ± 46.5	0.217	0.07	0.13	−0.75, 1.00
**Knee extensor endurance test**						
Time to task failure, sec	220.1 ± 65.9	221.1 ± 66.1	0.923	0.05	0.02	−0.86, 0.89
Mean torque output, Nm	46.8 ± 9.2	48.5 ± 9.1	0.139	0.08	0.18	−0.69, 1.06
Peak EMG of VL,% of MVC	33.2 ± 9.6	35.4 ± 14.3	0.642	0.08	0.18	−0.69, 1.06
Peak EMG of VM,% of MVC	29.7 ± 15.5	31.8 ± 10.9	0.623	0.07	0.16	−0.72, 1.03
Peak EMG of RF,% of MVC	25.2 ± 12.5	24.6 ± 6.3	0.857	0.05	−0.05	−0.93, 0.82

*Values are presented as mean ± standard deviation. CSA, cross-sectional area; MVC, maximum voluntary contraction; EMG, electromyography; VL, vastus lateralis; VM, vastus medialis; RF, rectus femoris*.

## Discussion

This study determined that a 2-weeks intervention of IPC did not increase whole-body exercise performance, as assessed by VO_2__peak_ during a ramp-incremental exercise test. This intervention also did not increase local exercise performance, as assessed by time to task failure during a sustained knee extensor endurance test. Moreover, the intervention did not change the dynamics of pulmonary VO_2_ and muscle deoxygenation during a repeated moderate-intensity exercise test. Furthermore, the intervention did not change the knee extensor MVC and quadriceps femoris CSA. These findings suggest that the 2-weeks IPC intervention may not be effective in increasing whole-body and local exercise performances and in improving its-related parameters. Therefore, the present findings could not corroborate the results of previous studies (Lindsay et al., 2017; Jeffries et al., 2018, 2019).

In a potential explanation of this discrepancy, previous studies employed an IPC intervention for 1 week (i.e., seven consecutive days) (Lindsay et al., 2017; Jeffries et al., 2018, 2019), whereas the present study employed an IPC intervention for 2 weeks (6 days/week). An acute bout of IPC increases circulating nitric oxide (NO) derived from endothelial NO synthase (Rassaf et al., 2014). The NO is a mediator for increasing whole-body exercise performance and skeletal muscle oxidative capacity (i.e., mitochondrial function) (Nisoli et al., 2004; Rassaf et al., 2007). Previous studies with supplementation of NO metabolite nitrate have determined that an increase in circulating NO induced by short-term nitrate supplementation increased whole-body and local exercise performances and skeletal muscle oxidative capacity (Bailey et al., 2009, 2010; Larsen et al., 2011). These previous studies employed intervention periods within 1 week (usually 3–6 days) (Bailey et al., 2009, 2010; Larsen et al., 2011). This reason can be at least partially explained by the inhibition of development of nitrate tolerance. Slysz and Burr (2019) reported that an 8-weeks IPC intervention was ineffective in increasing whole-body exercise performances, including $\dot{\text{V}}$O_2peak_, in endurance runners. Therefore, interventions longer than 1 week of IPC may be unfavorable for obtaining the positive effects of IPC on exercise performance and its-related parameters, potentially because of decreasing the efficacy of NO.

As another potential explanation, previous short-term intervention studies employed four episodes of a ischemia and reperfusion cycle per session (Lindsay et al., 2017; Jeffries et al., 2018, 2019), whereas the present study employed three episodes of this cycle based on our and other previous studies that reported positive effects of acute IPC on whole-body and local exercise performances (de Groot et al., 2010; Crisafulli et al., 2011; Kido et al., 2015; Paradis-Deschênes et al., 2016; Tanaka et al., 2016). In a review by Salvador et al. (2016), they suggested that the degree of positive effect of acute IPC on exercise performance may be comparable between three and four episodes of the ischemia and reperfusion cycle. Indeed, our previous studies have reported that acute IPC protocol consisting of the three episodes of the ischemia and reperfusion cycle increases endurance (i.e., time to exhaustion or task failure) during whole-body cycling and localized knee extensor exercise (Kido et al., 2015; Tanaka et al., 2016). Furthermore, this IPC protocol accelerated muscle deoxygenation dynamics of the exercising muscle (i.e., the vastus lateralis) during both exercises (Kido et al., 2015; Tanaka et al., 2016). Therefore, although the difference in the number of the ischemia and reperfusion cycle per session during short-term IPC intervention on exercise performance and its-related parameters remains unclear, the episode number may have a little effect on these variables.

Several studies have reported that short- and long-term IPC interventions (e.g., 1–8 weeks) of IPC increases endothelial function (Kimura et al., 2007; Nakamura et al., 2009; Jones et al., 2014, 2015). Of those, Jones et al. (2015) reported that endothelial function further increased a week after a 1-week intervention (i.e., seven consecutive days) compared to 24 h after the completion of this intervention. This phenomenon may be due to the late effect of IPC (Manchurov et al., 2014; Hildebrandt et al., 2016). Lindsay et al. (2017) reported that VO_2__peak_ further increased a week after a 1-week IPC intervention compared to 48 h after the completion of this intervention. In the present study, we did not evaluate the late effect of a 2-weeks IPC intervention. If this effect was examined in this study, we could be found the positive effects of short-term IPC intervention on exercise performance.

Previous studies have determined that short-term intervention (i.e., 2 weeks) of blood flow restriction, performed with a similar protocol (e.g., occlusion pressure) to that of IPC, mitigated strength loss and muscle atrophy in subjects with disuse muscle atrophy due to immobilization and orthopedic surgeries (Takarada et al., 2000; Kubota et al., 2008). Despite the underlying potentiality on muscle strength and size adaptations, no study had examined the effects of short-term IPC intervention on muscle strength gain and hypertrophy prior to the present study. Unfortunately, we determined that a 2-weeks IPC intervention did not increase the knee extensor MVC and quadriceps femoris CSA. Although this study could not detect the positive effects of short-term IPC intervention on muscle strength and size, this is the first study to evaluate these muscle adaptations.

In this study, we recruited only healthy young adults; thus, whether the present findings can be generalized to other age groups (e.g., older individuals) and health state populations (e.g., patients with chronic diseases) remains unclear. The IPC intervention is generally more required in older individuals and patients with chronic diseases than in healthy young adults due to their deteriorating cardiovascular and musculoskeletal systems, which may be major limiting factors in performing effective exercise training programs to improve exercise performance. Pryds et al. (2017) reported that a 4-weeks intervention of remote IPC (i.e., applied to upper arm) increased the knee extensor strength in older individuals and patients with chronic ischemic heart failure; however, the effect of local IPC intervention on muscle strength in these populations has not yet been elucidated. Further studies are needed to determine the effect of short-term IPC intervention on exercise performance in various populations.

Paradis-Deschênes et al. (2020) reported that IPC combined with sprint-interval training throughout 4 weeks (2 days/weeks) increased exercise performance and its-related parameters during 5-km time trial (e.g., completion time) and 30-s Wingate test (e.g., fatigue index) more than those of placebo intervention (i.e., training alone) in endurance athletes. They also determined positive effects of the IPC intervention on perfusion and metabolic changes (e.g., changes in deoxy-hemoglobin/myoglobin) of the exercising muscle during these whole-body exercises (Paradis-Deschênes et al., 2020). Additionally, Carvalho et al. (2020) reported that IPC combined with knee extensor resistance training throughout 6 weeks (2 days/week) increased the knee extensor one-repetition maximum more than the placebo intervention in resistance-trained individuals. Furthermore, Surkar et al. (2020) reported that remote IPC combined with wrist extensor resistance training throughout 2 weeks (3 days/weeks) increased the wrist extensor one-repetition maximum more than the placebo intervention in healthy young adults. These findings suggest that a combination intervention of IPC and exercise training, rather than IPC intervention alone, may be more effective to increase whole-body exercise and its-related parameters. Therefore, further investigations on the effective protocols of short-term IPC intervention combined with exercise training on exercise performance would be beneficial.

This study had several limitations. First, the statistical values for the measured parameters in this study showed insufficient statistical power and sensitivity. This is mainly associated with small sample size (i.e., 10) of this study. A meta-analysis by Salvador et al. (2016) reported that the positive effect of acute IPC on exercise performance had a small effect (i.e., effect size = 0.43). When considering the close interaction between the effects of an acute bout and short-term intervention, if this effect size can be utilized for assessing a necessary sample size of short-term intervention, a considerable number of subjects is required for the intervention study. Thus, to obtain sufficient statistical power and sensitivity, a larger number of subjects than that of this study would be essential, which is a major limitation. Additionally, this study did not include control group(s) with placebo and/or nocebo, which is also a major limitation. Altogether, further studies with a larger sample size and control group(s) are required to clarify the present findings.

## Conclusion

This study determined that a 2-weeks intervention of IPC did not increase whole-body and local exercise performances, corresponding with ineffectiveness on its-related parameters in healthy young adults. Therefore, we suggest that the short-term IPC intervention may have little or no effect on exercise performance. Nevertheless, because this study has several limitations, further studies may be required to reconfirm the effect of short-term IPC intervention on exercise performance with adequate and rigorous methodology.

## Data Availability Statement

The raw data supporting the conclusions of this article will be made available by the authors, without undue reservation.

## Ethics Statement

The studies involving human participants were reviewed and approved by the Ethics Committee of Ritsumeikan University. The patients/participants provided their written informed consent to participate in this study.

## Author Contributions

DT and TS conceived, designed the experiment, and wrote the manuscript. DT, TS, and KS performed experiments. DT, TS, KS, and TI interpreted results of experiments. TS and TI edited and revised manuscript. All authors have read and approved the manuscript.

## Conflict of Interest

The authors declare that the research was conducted in the absence of any commercial or financial relationships that could be construed as a potential conflict of interest.
